# Evaluating the Status of and African Wild Dogs *Lycaon pictus* and Cheetahs *Acinonyx jubatus* through Tourist-based Photographic Surveys in the Kruger National Park

**DOI:** 10.1371/journal.pone.0086265

**Published:** 2014-01-21

**Authors:** Kelly Marnewick, Sam M. Ferreira, Sophie Grange, Jessica Watermeyer, Nakedi Maputla, Harriet T. Davies-Mostert

**Affiliations:** 1 Endangered Wildlife Trust, Johannesburg, South Africa; 2 Centre for Wildlife Management, University of Pretoria, Pretoria, South Africa; 3 Scientific Services, SANParks, Skukuza, South Africa; 4 Wildlife and Reserve Management Research Group, Zoology and Entomology Department, Rhodes University, Grahamstown, South Africa; 5 African Wildlife Foundation, Nairobi, Kenya; 6 Department of Zoology, Wildlife Conservation Research Unit, Recanati-Kaplan Centre, Oxford University, Oxford, United Kingdom; University of Regina, Canada

## Abstract

The Kruger National Park is a stronghold for African wild dog *Lycaon pictus* and cheetah *Acinonyx jubatus* conservation in South Africa. Tourist photographic surveys have been used to evaluate the minimum number of wild dogs and cheetahs alive over the last two decades. Photographic-based capture-recapture techniques for open populations were used on data collected during a survey done in 2008/9. Models were run for the park as a whole and per region (northern, central, southern). A total of 412 (329–495; SE 41.95) cheetahs and 151 (144–157; SE 3.21) wild dogs occur in the Kruger National Park. Cheetah capture probabilities were affected by time (number of entries) and sex, whereas wild dog capture probabilities were affected by the region of the park. When plotting the number of new individuals identified against the number of entries received, the addition of new wild dogs to the survey reached an asymptote at 210 entries, but cheetahs did not reach an asymptote. The cheetah population of Kruger appears to be acceptable, while the wild dog population size and density are of concern. The effectiveness of tourist-based surveys for estimating population sizes through capture-recapture analyses is shown.

## Introduction

African wild dogs *Lycaon pictus* and cheetahs *Acinonyx jubatus* are threatened throughout their range and the Kruger National Park (hereafter Kruger) and its neighbouring conservation areas represent an essential core area for their conservation [1] [2]. Both species are sub-dominant members of the African large carnivore guild with lions *Panthera leo* and spotted hyaenas *Crocuta crocuta* being dominant over them through exploitive competition [3] [4]. Additionally, cheetahs and wild dogs have large space requirements and thus occur at low densities [4] [5] even in large protected areas [6].

Small populations pose conservation challenges for two key reasons. Firstly, extinction risk in small populations is potentially higher since it is mainly driven by demographic and environmental stochastic effects and random catastrophes [7]. Secondly detecting trends and thus local extinction risks in small populations is statistically challenging [8].

The wild dog population in Kruger has been monitored using photographic surveys in 1988/9 (survey period June 1988–June 1989) [9], 1994/5 (survey period June 1994–June 1995) [10], 1999/2000 (survey period May 1999–June 2000) [11] and 2004/5 (survey period October 2004–April 2005) ([12]) with cheetah being included in the surveys during 1990/1 [13] and 2004/5 [12]. All photographic surveys gave an estimate of the minimum number of animals alive on 1 January of the survey period [9]. While this was a useful measure of the status of the population, more robust methodologies now can be applied to photographic data to obtain more accurate population estimates with confidence intervals.

This study assessed the status of cheetahs and wild dogs in Kruger using capture-recapture models applied to data obtained from tourist photographic surveys. The survey intensities necessary for obtaining reliable population estimates were determined to help inform effective future monitoring systems.

## Materials and Methods

### Study Area

**Figure 1:**
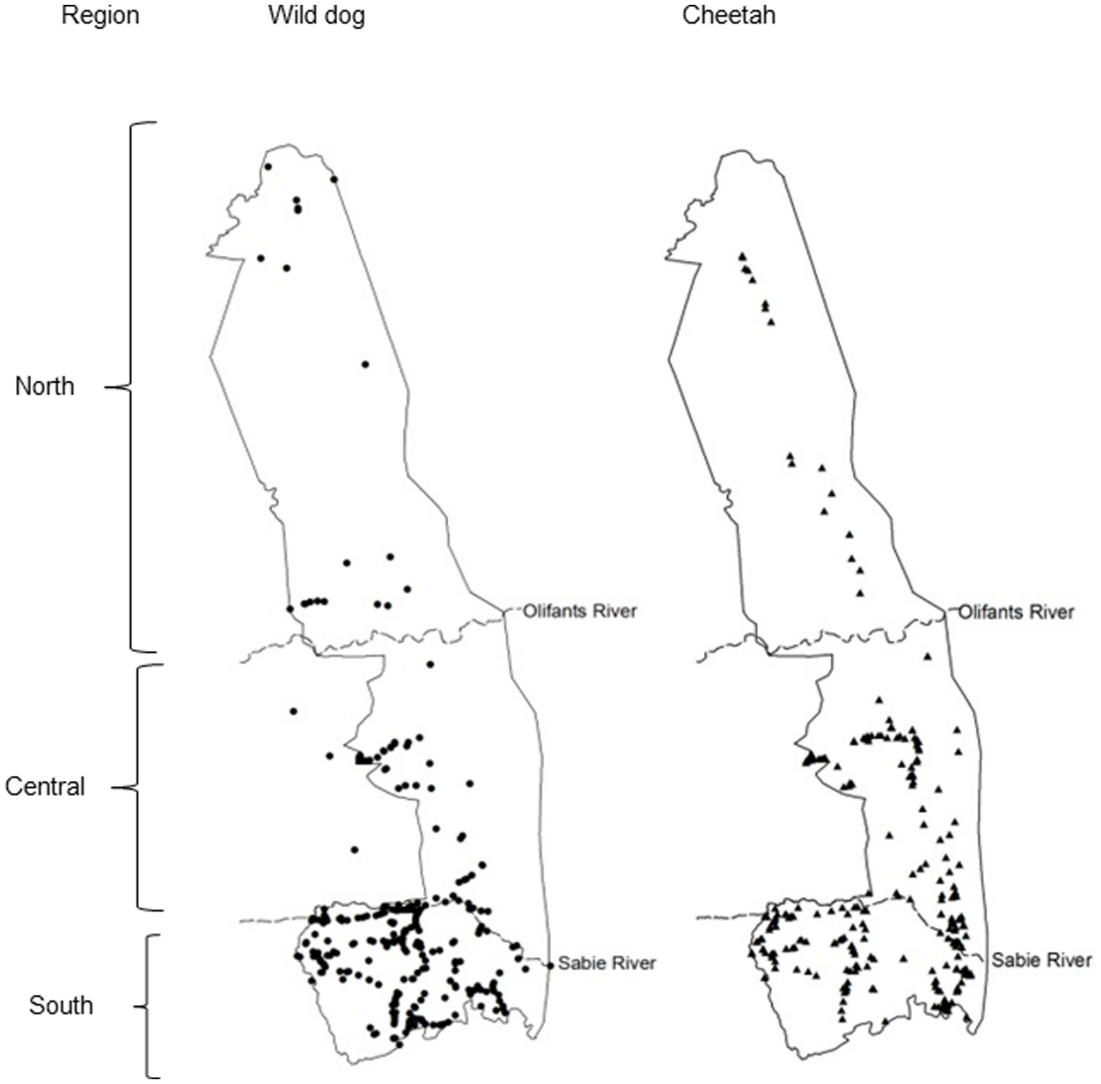
Wild dog and cheetah sightings in the Kruger National Park during the 2008/9 tourist photographic survey. The regions for analysis are delineated as follows: southern = south of the Sabie River, central = between Sabie and Olifants Rivers, northern = north of the Olifants River.

The study was conducted in the 21353 km^2^ Kruger National Park and neighbouring private reserves in South Africa with permits issued by SANParks under registered research project number DMOHT582. Field work and advertising were restricted to the Kruger National Park, thus no permits or permissions were required to obtain entries from neighbouring areas. The analysis was based on three separate regions: southern region (south of the Sabie River); central region (between the Sabie and Olifants rivers) and northern region (north of the Olifants River) (Fig. 1). These were defined by differences in prey biomass [14] and tourist numbers [9] which can collectively lead to variations in carnivore density, frequency of observation and detection. There is a decrease in gradient from south to north in prey biomass, density of roads and infrastructure, and tourist volumes. These differences can lead to variations in sample effort in both time and space.

### Data Collection

The latest tourist photographic survey for cheetahs and wild dogs was done from 1 July 2008 to 30 April 2009 using methodology following Maddock & Mills [9]. Wild dogs breed annually at mid-year making this a good time to estimate wild dog numbers. Cheetahs breed aseasonally making survey timing irrelevant. During this time, tourists and park staff were asked to submit sighting details to the project with photographs, dates and locations. The survey was promoted through a photographic competition, and flyers and posters were distributed throughout the park at gates and camps. A web site was developed and several local radio adverts were broadcast. A Census Hotline Number was established that tourists could text to report sightings of cheetahs or wild dogs that could be followed up by a field worker. Promotional material was actively distributed to tourists and staff to encourage submission. Entries were received by e-mail, post and by hand. All animals photographed were identified using their unique pelage patterns. Locations were georeferenced using the description given by the entrant.

### Sampling Effort

To investigate possible differing detection rates between regions, the number of day visitors and tourist bed nights occupied in each camp were collated (data provided by South African National Parks). Where access gates or rest camps were located on the boundaries defining the three regions, half of the bed nights and day visitors were assigned to each region. The average daily number of visitors was calculated at weekly intervals.

To determine the relationship between population estimates and tourist-related effort, an effort index was developed which scaled tourist volumes to the area and road density in each region (n/km/km^2^). This index was plotted against the accumulation of newly identified individual animals to define an accumulation curve described by the negative exponential model (y = a[1–e^−bx^]). The derivative of this model allowed for estimating the effort at which new individuals were recorded, less than 0.1 individuals added per unit of increasing tourist effort was considered evidence that an asymptote had been reached.

The number of entries was predicted to increase with time as awareness of the photographic competition increased. Entries were thus related to weeks into the competition using non-linear curve fitting. To evaluate the assumption that more observers lead to more observations, the residual values for entries were calculated to remove the effect of time on entries and these were related to the average daily number of tourists present in that week. A tourist-related effect on sampling was concluded if this linear relationship was significant (p<0.05).

### Population Estimates

The data for cheetahs and wild dogs were prepared for capture-recapture analyses by using all captures for the period 1 July 2008 to 30 April 2009. The data were collapsed to form 10 capture occasions where, one month was equated to one capture period. Thus any animal photographed at any time during that month was considered captured during that month.

Life histories were compiled for each identified individual photographed and consisted of 10 occasions of capture coded as “1” for a captured individual and “0” for a non-captured individual. Each individual was assigned to a region of the park based on the majority of recorded sightings, allowing for regional population estimates.

Goodness-of-fit (GOF) tests were run in U-CARE [15] to detect potential problems in the structure of the data files. The appropriate data files were selected and used to run open capture-recapture models (POPAN) in MARK [16] to estimate population sizes of cheetahs and wild dogs.

For cheetahs, adult males are associated with each other in small coalitions (2–3 individuals), whereas each adult female is associated with her cubs. For wild dogs, animals of all ages are associated with specific packs. These social structures are likely to result in heterogeneity in in individual life histories; because individuals in the same social group are more likely to be captured during the same occasion than individuals in other groups. This violates the assumption of the capture-recapture models that all individuals in a population have equal capture probabilities. To account for this, sub-sets of the data were built which took the social structure of the species into account.

Firstly, datasets were built at a park-wide scale (i.e. data from all three regions were used). For cheetahs, the datasets including single adult males, adult females and adult unknowns could not be used because the data structure was not suitable (GOF test: p = 0.02). A dataset including 145 adults and sub-adults classified by sex (male, female, unknown) was selected for cheetahs (GOF test: p = 0.82). No cheetah cubs were included in the analyses because cubs are always associated with their mothers. These animals were accounted for by estimating the mean size of family groups (one female and her offspring) (4.87±0.44 SE; n = 15) to calculate the total number of adult females and cubs in the population. The final number of females from the capture-recapture estimate was multiplied by the mean female group size and added to the population estimate to produce a result that accounted for these groups.

For wild dogs each pack was used for the capture-recapture modelling i.e. if an individual in a specific pack was captured, the whole pack was considered captured. This resulted in the selection of a dataset that included 21 packs (155 wild dogs with all age- and sex-classes combined) (GOF test: p = 0.14). Capture-recapture models were used to estimate the total number of packs in Kruger. The mean pack size was then estimated (7.4±1.3 SE; n = 21) and multiplied by the number of packs from the capture-recapture modelling to estimate the total number of wild dogs in the population.

Secondly, datasets were built for each region (three per species: northern, central and southern regions). For cheetahs, datasets that included adults and sub-adults classified by sex (male, female, unknown) were selected (central region: n = 53, GOF test: p = 0.93; southern region: n = 79, GOF test: p = 0.98). The sample size for the northern region (n  = 13) was not sufficient to run GOF tests. For wild dogs, a dataset including 21 packs classified into three regions was selected (GOF test: p = 0.70).

Finally, POPAN models using selected datasets for the park and for each region were run. In each instance, the model selected had the lowest Akaike Information Criterion (AICc for small sample size) and lowest number of parameters [17].

### Population Characteristics

Population structures for cheetahs and wild dogs were determined from photographs. Wild dogs were assigned to adult/yearling (>1 year old) and pup (<1 year old) age classes for males, females and animals of unknown sex. Cheetahs were assigned to cubs and adult male, female and unknown. Capture-recapture models were able to be used to determine the abundance of the three adult sex classes for cheetahs. Due to the dependency of capture probabilities between pack members, wild dog age and sex structure could not be determined using capture-recapture models; instead counts using the photographic records were used.

### Optimal Survey Intensity

Optimal survey intensities were determined by calculating a series of population estimates using mark-recapture, with the associated confidence intervals, from sub-samples of entries, ranging from 15 entries to the complete datasets for both species. Each confidence interval was expressed as a percentage of the estimate, i.e. a percentage confidence limit (PCL) (PCL = 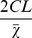) [18]. PCLs of 20% typically translate to a coefficient of variance of ≈5% while those of 40% translate to ≈10%. The number of entries required to produce population estimates with CVs of ≈5% and ≈10% were determined using the fitted equation y = 1.558×^−0.373^ for wild dogs and y = 1.464×^−0.212^ for cheetahs where y = PCL and x = number of entries.

## Results

### Data Collection and Sampling Effort

**Figure 2:**
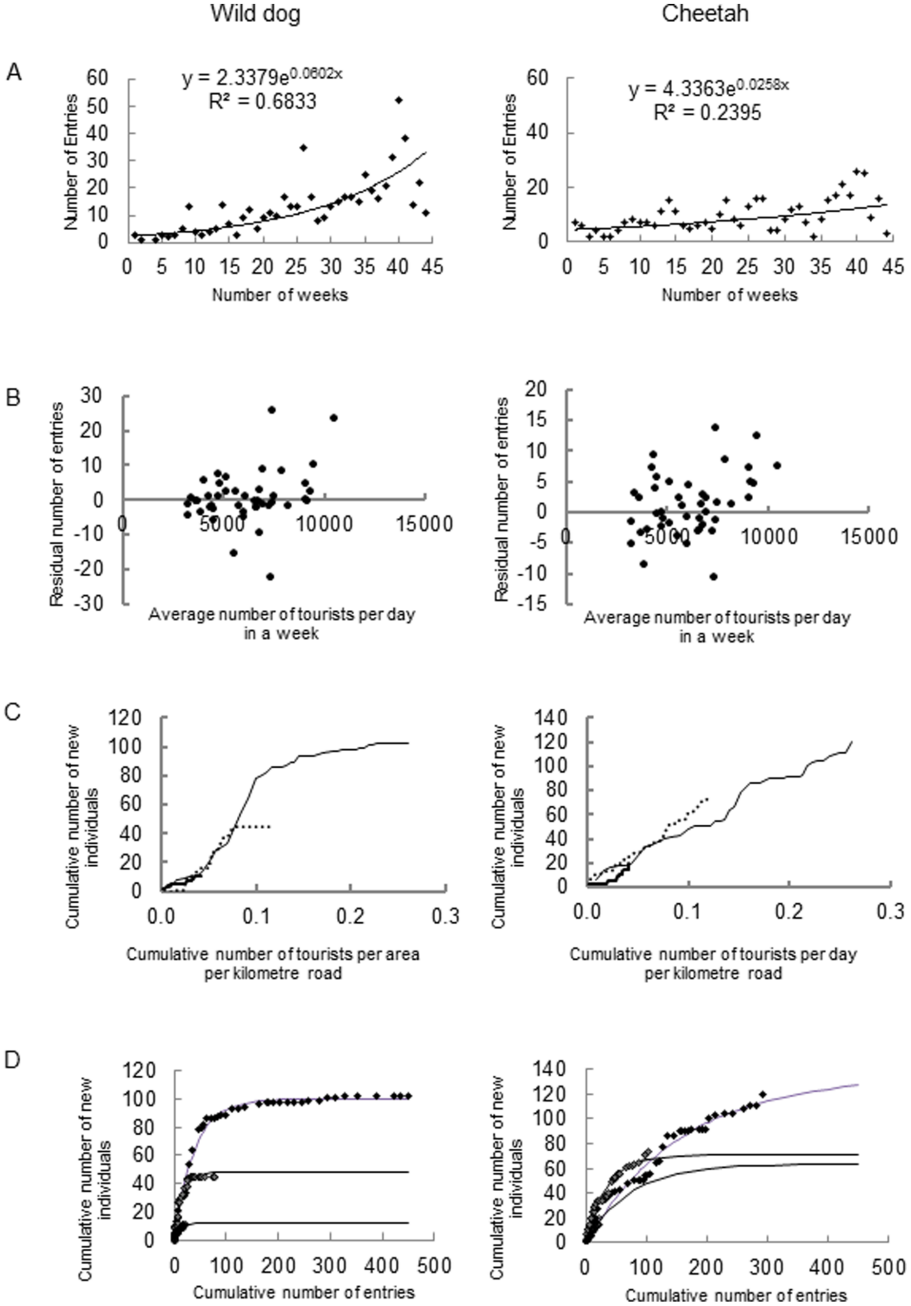
Sampling effort in the 2008/9 Kruger National Park tourist photographic survey of cheetahs and wild dogs. A: The weekly number of entries received over time. B: The relationship between the weekly number of entries and available tourists during that time - effect of time removed. C: Accumulation of new individuals as the number of tourists per area and available roads in a region increases. (northern – solid line, central – broken line, southern – solid thin line. D: Accumulation of new individuals as the number of entries increases (northern – open symbols, central – shaded symbols, southern – solid symbols).

The number of photographic entries varied over time and between regions with a general trend of more entries being received from the southern regions (Table 1). The number of entries per week for both species increased exponentially over time (Fig. 2). The number of wild dog entries was not associated with the number of tourists once the effect of time was accounted for (F_1,42_ = 4.03, p = 0.06; Fig. 2B) while the number of cheetah entries increased as tourist numbers increased (F_1,42_ = 6.02, p = 0.02; Fig. 2B).

**Table 1:** Population estimates of cheetahs and African wild dogs derived from POPAN models in MARK.

	Cheetahs					Wild dogs				
Parkregion	Numberof entries	POPANModel	Estimate	SE	95%CI	Numberof entries	POPANModel	Estimate	SE	95%CI
North	24	NA^1^	NA^1^	NA^1^	NA^1^	24	φ(g*t) p(g) β(t) N(g)	24	1.60	19–29
Central	107	φ(.) p(g) β(g*t) N(g)	137	26.72	83–191	89		23	1.15	20–27
South	312	φ(g) p(g) β(t) N(g)	236	31.24	174–298	450		89	0.91	87–91
Total	454^2^	φ(i) p(g*t) β(t) N(g)	412	41.95	329–495	564^3^	φ(i) p(i) β(t) N(i)	151	3.21	144–157

Data collected through a tourist photographic survey during 2008–2009 with the number of entries received per region displayed.

1 Sample size insufficient.

2 1 unknown region.

3 11 unknown region.

In all three study regions, the accumulation of new wild dogs per unit effort reached asymptotes i.e. less than 0.1 individuals added per unit of increasing tourist effort (Fig. 2C, Northern: y = 26.99[1–e^−11.44x^], R^2^ = 0.82; Central y = 243.88[1–e^−2.04x^], R^2^ = 0.91; Southern: y = 135.06[1–e^−6.57x^], R^2^ = 0.92). For cheetahs, no asymptotes were reached (Fig. 2C, Northern: y = 8523.92[1–e^−0.04x^] R^2^ = 0.80; Central: y = 2844.42[1–e^−0.21x^], R^2^ = 0.99; Southern: y = 533.54[1–e^−0.97x^], R^2^ = 0.98).

The rate of accumulation of new wild dogs decreased with increasing entries (Fig. 2D, Southern: y = 99081[1–e^−0.03x^], R^2^ = 0.99; Central: y = 48.08[1–e^−0.06x^], R^2^ = 0.97; Northern: y = 12.20[1–e^−0.09x^], R^2^ = 0.83). Less than 10% new wild dog additions per week were obtained at 126, 56 and 28 entries in the southern, central and northern regions, respectively.

The rate of accumulation of new cheetahs decreased with increasing entries (Fig. 2D, Southern: y = 134.76[1–e^−0.01x^], R^2^ = 0.96; Central: y = 71.14[1–e^−0.03x^], R^2^ = 0.97; Northern: y = 63.43[1–e^−0.01x^], R^2^ = 0.98). Less that 10% new individuals per week were obtained at 157, 105 and 338 entries from the south, central and northern, respectively.

### Population Estimates

#### Cheetahs

For the whole park, the selected model included a group (sex) effect on the estimated population size, with 94 (±6.66 SE) adult males, 38 (±4.49 SE) adult females and 134 (±35.18 SE) unknown adults estimated. In total, the number of adult cheetahs was estimated at 266 individuals in Kruger as a whole. Using the average size of cheetah families (4.867±0.435 SE; N = 15), the total population size of cheetahs in Kruger was therefore estimated at 412 individuals (Table 1). There was no estimate of population size for the northern region since the sample size was too small to run models.

For the central region, the selected model included a group (sex) effect on the estimated population size, with 28 (±3.84 SE) adult males, 7 (±1.62 SE) adult females and 74 (±25.25 SE) unknown adults. The total number of adult cheetahs was estimated at 110 individuals in the central region. Using the average size of cheetah groups (4.867±0.435 SE; N = 15), the total population size of cheetahs in the Central region was estimated at 137 individuals (Table 1).

For the southern region, the selected model included a group (sex) effect on the estimated population size, with 57 (±5.45 SE) adult males, 26 (±3.20 SE) adult females and 52 (±26.51 SE) unknown adults. The total number of adult cheetahs was estimated at 135 individuals in the southern region. Using the average size of cheetah groups (4.867±0.435 SE; N = 15), the total population size of cheetahs in the southern region was estimated at 236 individuals (Table 1).

#### Wild dogs

For the whole park, the selected model did not include any effect on the estimated population size, with 20 (±0.44 SE) packs. The total number of wild dog packs was estimated at 20 packs in the whole of Kruger. Using the average pack size (7.381±1.343 SE; n = 21), the total population size of wild dogs in Kruger was estimated at 151 individuals (Table 1).

When regions were considered, the selected model included a regional effect on the estimated population size with three (±0.22 SE) packs in the northern region, three (±0.16 SE) packs in the central region and 12 (±0.12 SE) packs in the southern region. Using the average size of a pack (7.381±1.343 SE; N = 21), the total population size of wild dogs was estimated at 23 individuals in the central region, 24 in the northern region and 89 in the southern region (Table 1).

### Population Characteristics

Capture–recapture models enabled the determination of the adult cheetah sex structure per region with the exception of the northern region where the sample size was too small to run the models (Table 2). Cheetah estimates are biased towards males for the sexed adults. Wild dog sex ratios from photographic counts were near parity for the whole park (Table 3).

**Table 2:** Population estimates of cheetahs in the different regions of the Kruger National park derived from POPAN models in MARK.

	Adult male			Adult female			Adult unknown			POPAN model	Total	
ParkRegion	Populationestimate	SE	95%CI	Populationestimate	SE	95%CI	Populationestimate	SE	95%CI		Adults	Allages
North	NA^1^	NA^1^	NA^1^	NA^1^	NA^1^	NA^1^	NA^1^	NA^1^	NA^1^	NA^1^	NA^1^	NA^1^
Centre	28	3.84	21–36	7	1.62	4–40	74	25.25	25–124	φ(i) p(g) b(g*t) N(g)	110	137
South	57	5.45	46–67	26	3.2	20–32	52	26.51	0–104	φ(g) p(g) b(t) N(g)	135	236
Total	94	6.66	81–107	38	4.49	29–47	134	35.18	65–203	φ(i) p(g*t) b(t) N(g)	266	412

Data collected through a tourist photographic survey during 2008–2009.

1 Sample size insufficient.

**Table 3:** Population estimates of African wild dogs in the different regions of the Kruger National park derived from count data collected through a tourist photographic survey during 2008–2009.

	Adult				Pup				Total
Park region	Male	Female	Unknown	Total	Male	Female	Unknown	Total	All ages
North	3	6	0	9	1	2	0	3	12
Central	10	11	4	25	5	12	2	19	44
South	29	35	6	70	9	12	9	30	100
Total	42	52	10	104	15	26	11	52	156

### Optimal Survey Intensity

**Figure 3:**
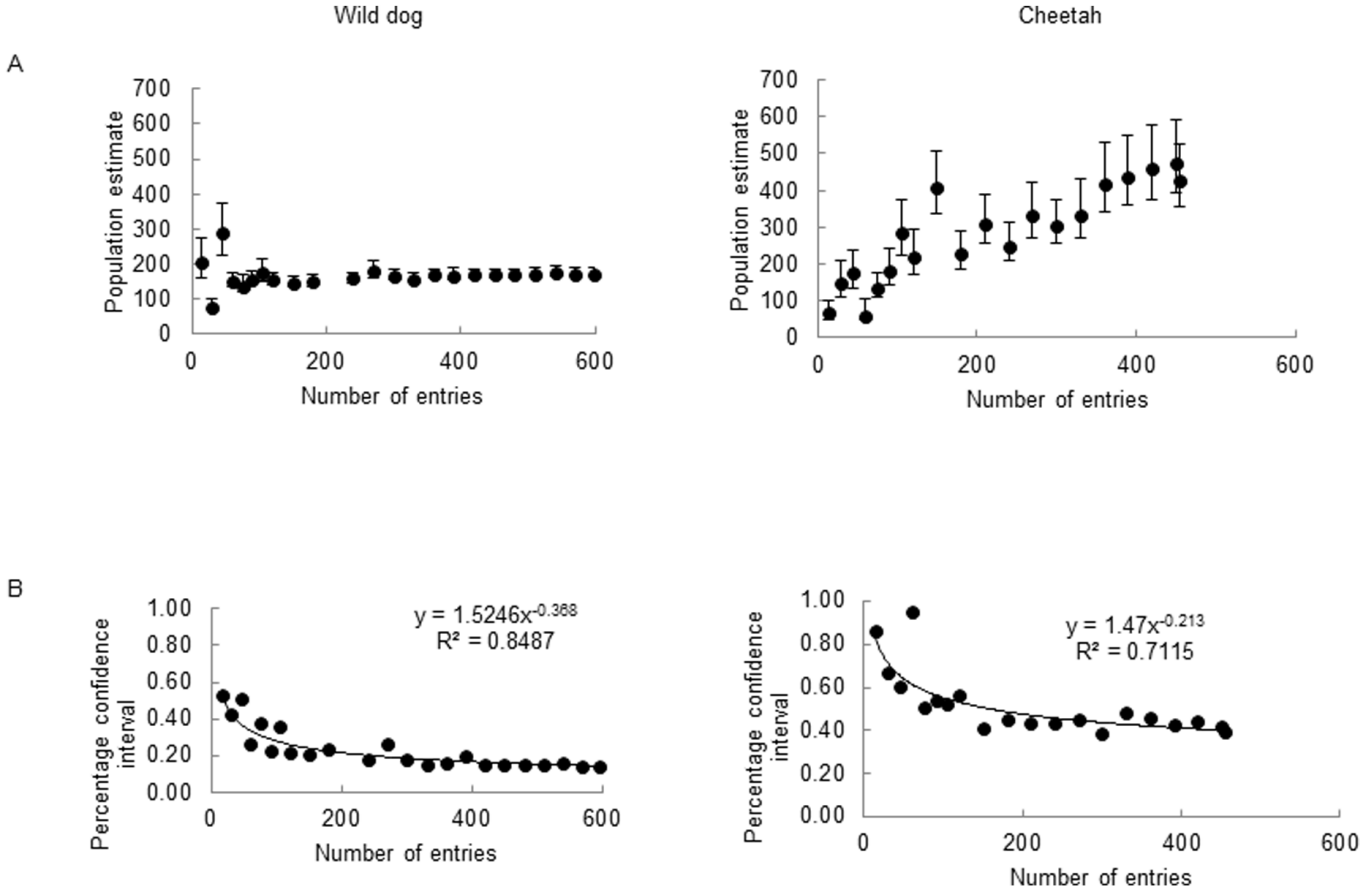
Effect of the number of entries on estimates. Population estimate (A) and percentage confidence intervals (B) for wild dogs and cheetahs in the Kruger National Park using tourist photographic surveys.

PCLs of estimates declined with increasing numbers of entries for wild dogs (R^2^ = 0.848) and cheetahs (R^2^ = 0.711) (Fig. 3). Wild dogs required 250 and 38 entries to return 20% and 40% PCLs, respectively (i.e. CVs of ≈5% and ≈10%). For cheetahs an unrealistic 11670 entries were required to return 20% PCL; a more achievable 451 entries were required for 40% PCL.

## Discussion

### Effectiveness of Tourist Photographic Surveys for Monitoring Wild Dogs and Cheetahs

Estimating population sizes for sub-dominant carnivore guild members is challenging both statistically and logistically. Photographic-based surveys have been used for several species [19] with capture-recapture estimates being applied when species have distinct pelage patterns, like cheetahs [20] and wild dogs. Public participation in photographic-based surveys is less used, but can generate data suitable for capture-recapture analyses.

Generating sufficient data through tourist-based surveys is integral to ensuring sampling success. In this survey, the number of photographic entries was sufficient to generate a reliable estimate for wild dogs and cheetahs at a park-wide scale and per park region except for the northern region for cheetahs. However, the wild dog population estimates from capture-recapture models had lower standard errors suggesting more effective sampling for wild dogs than for cheetahs.

Analysis of results from public-generated data present challenges through biases introduced through a lack of control over survey effort and area. This makes survey effort difficult to measure and data may be biased towards areas with higher visitation rates. In this study, survey effort was not uniformly distributed with a higher density of tourists and roads in the south which decreased in a gradient towards the north. This can lead to variations in capture probability which can affect the outcomes of the capture-recapture models. However, this was accounted for by dividing the study area into the three separate regions (northern, central, southern) based on differences in tourist volumes, infrastructure and prey density.

Individual capture-recapture models were run for each region separately to account for these spatial differences across the study area. For wild dogs the regional models showed that the capture probability varied by region meaning that some of these spatial differences could be affecting the survey for wild dogs, but not for cheetahs.

The selected cheetah capture-recapture model for the whole park showed that the capture probability of cheetahs was dependent on time i.e. the number of entries, but this was not relevant at the regional level or for any of the selected wild dog models. This means that capture probability was not affected by time or number of entries for any of the selected models, except for cheetahs at a park-wide scale.

The number of entries received was not influenced by the number of tourists, but was most likely associated with the chance of encountering animals. The higher number of entries in the southern region is probably a consequence of larger population sizes for both species in this region.

While there are more cheetahs (n = 412) than wild dogs (n = 151) in the park, more entries were received for wild dogs than cheetahs. This difference may be related to social behaviour. Cheetah females occur as singletons, unless with cubs, and males either singly or in coalitions comprising two to three individuals [21]. This could lead to cheetahs being less detectable than wild dogs which occur in large packs. Group size also affects detection probabilities for other species like feral horses [22]. Additionally, wild dogs are more wide-ranging than cheetahs and frequently use roads to traverse large distances which could make them more detectable than cheetahs. Wild dogs are rarer than cheetahs and it may also be possible that tourists are more excited about viewing them than cheetahs and thus more likely to submit wild dog entries to the competition.

### Survey Intensity

In this study, it was more difficult to obtain precise population estimates for cheetahs than for wild dogs through tourist surveys. More than 11000 cheetah entries are required to achieve estimates with PCLs of 20% while wild dogs require only 250 entries. Thus, it is more feasible to aim at obtaining cheetah estimates with PCLs of 40% for which approximately 450 entries are required.

### Population Status

The male-biased sex ratio of cheetahs in Kruger is potentially an artefact of the survey method. Males are probably easier to sex from photographs than females due to the former’s external genitalia. Additionally, male cheetahs are probably more detectible than females because they occur in coalitions [22], use roads and prefer more open habitat [23]. This trend was confirmed by the selected capture-recapture models for cheetahs at the park-wide scale that showed the probability of cheetah capture varied with sex for male, female, unknown sex models. The observed patterns in the sex structure of cheetahs in Kruger are therefore likely to be a result of limitations of the survey method and animal behaviour rather than biological effects that would suggest consequences for their conservation status.

Wild dog sex ratios are near parity as would be expected. The effect of sex could not be tested using capture-recapture models due to packs being used in the models and not individuals. However, it makes biological sense that wild dogs of both sexes would have similar capture probabilities. Wild dogs live in packs and the behaviour of males and females is not different enough to affect capture probabilities as it does for cheetahs.

The estimated cheetah population of 412 individuals translates to a density of approximately 0.193 cheetahs/km^2^ in the whole of Kruger. While there are no appropriate historical data to compare this estimate to, in other areas cheetahs have been recorded at lower densities of 0.016–0.044/km^2^ in the Serengeti [24] and 0.009–0.102 cheetahs/km^2^ in Kenya [25]. From these estimates, there is currently no reason for conservation concern around the Kruger cheetah population.

The estimated wild dog population of 151 individuals in 20 packs translates to a density of approximately 0.007 wild dogs/km^2^ in the whole of Kruger. This is low in comparison to historical data when in 1994 an estimated minimum count of 357 wild dogs (0.017 wild dogs/km^2^) in 26 packs was recorded [9]. In other protected areas wild dogs occur at densities of varying between 0.015 in Hwange to 0.040 in the Selous [3]. This small size and apparent declining nature of the Kruger wild dog population is of concern as this is South Africa’s largest protected population and one of the key populations in Africa. This needs to be further investigated.
