# Correction: Evaluating the Status of and African Wild Dogs Lycaon pictus and Cheetahs Acinonyx jubatus through Tourist-based Photographic Surveys in the Kruger National Park

**DOI:** 10.1371/journal.pone.0091493

**Published:** 2014-02-28

**Authors:** The PLOS ONE Staff

The article Title is incorrect. The correct Title is: “Evaluating the Status of African Wild Dogs *Lycaon pictus* and Cheetahs *Acinonyx jubatus* through Tourist-based Photographic Surveys in the Kruger National Park.”

The correct Citation is: Marnewick K, Ferreira SM, Grange S, Watermeyer J, Maputla N, et al. (2014) Evaluating the Status of African Wild Dogs *Lycaon pictus* and Cheetahs *Acinonyx jubatus* through Tourist-based Photographic Surveys in the Kruger National Park. PLoS ONE 9(1): e86265. doi:10.1371/journal.pone.0086265
